# Idiopathic Macular Hole: A Comprehensive Review of Its Pathogenesis and of Advanced Studies on Metamorphopsia

**DOI:** 10.1155/2019/7294952

**Published:** 2019-05-23

**Authors:** Qi Chen, Zao-xia Liu

**Affiliations:** Department of Ophthalmology, The Second Hospital of Jilin University, Changchun, China

## Abstract

Vitreous anteroposterior traction is an important factor that affects macular hole (MH) formation at the early stage, and vitreous tangential traction can lead to further hole expansion after hole formation. Recent studies have highlighted the significance of Müller cells for the pathogenesis of MH. Since the advent of MH treatment, success rates for MH closure have significantly improved, as has postoperative visual acuity. However, metamorphopsia, an initial and common symptom of MH, still exists. Metamorphopsia is significantly related to the deterioration of visual quality of life and can be used as an independent index to evaluate visual function before and after surgery. In MH patients, metamorphopsia has different manifestations representing different clinical implications. M-CHARTS, as a new means of inspection, can quantify the degrees of metamorphopsia, and with the development of optical coherence tomography (OCT), layer-by-layer scanning of the retinal structure has become possible. These methods enable detailed analysis of the connections between the degree of metamorphopsia and relevant OCT parameters. Preoperative OCT parameters can be used to evaluate the prognosis of the postoperative visual function of MH patients and are therefore of great significance in guiding the treatment of MH patients.

## 1. Introduction

Idiopathic macular hole (IMH), as a kind of common macular diseases, involves tissue defects including the retinal internal limiting membrane (ILM) and even the photoreceptor (PR) layer [1], and it is known that vitreous traction on local retina, posterior vitreous detachment (PVD) around the macular fovea, and continuous adhesion are major etiological factors in most IMH cases [1–4]. IMH typically affects elderly patients, and approximately two-thirds of patients are females [5]. The main clinical manifestations of IMH include decreased vision, metamorphopsia, and central dark spots, which can occur suddenly or gradually. The incidence of MH among the common population has been reported to range from 0.2% to 0.8% [4, 6]. To understand the pathogenesis of macular hole in relation to metamorphopsia, we must first clarify the pathogenesis of macular hole, that is, the whole process from the impending macular hole to the complete formation of the macular hole. Secondly, it is necessary to explore the related research of metamorphopsia. In this way, the study metamorphopsia of macular holes can be fully understood.

## 2. Several Hypotheses on the Development of MH

### 2.1. Vitreous Macular Traction in the Pathogenesis of IMH

Among the hypotheses concerning the pathogenesis of IMH, the most extensively accepted one is the exertion of direct A-P (anterior-posterior) traction by the posterior vitreous cortex on the macular fovea [1–3, 7, 8]. In healthy human eyes, the vitreous humor of the posterior vitreous cortex slowly passes through the optic disc, which realizes the separation of the posterior vitreous cortex and the posterior pole. In contrast, in human eyes at the risk of IMH formation, abnormal vitreous macular adhesions produce dynamic traction, and the contraction of collagen fibers in the longitudinal direction causes progressive anterior traction until avulsion of the Müller cap occurs [9–11]. According to recent research on the early stage of IMH formation, the strength of A-P traction was higher when the area of perifoveal vitreous detachment was smaller. However, tangential traction could not have an effect until the IMH had formed. Tangential traction is formed when the residual vitreous remains on the fovea after the PVD contracts [12], during which Müller cells proliferate and invade the ILM. When the proliferating glial cells on the ILM shrinks, the hole is enlarged [13–17].

Studies on the visual distortion of macular holes emphasize the role of the photoreceptor cell layer, which is the origin of the initial stage of the signal transduction pathway. The above exposition on the pathogenesis of the macular hole is from only the macroscopic level. Although this perspective emphasizes the role of the vitreous traction in the fovea, it does not allow for studies of occurrence and development of the macular hole at the cellular and molecular levels. To elucidate the cause of MH visual distortion at the cellular level, further study of the pathogenesis of macular holes is needed. Therefore, with the latest research on Müller cells in the macular, we will have a clearer understanding of the whole process of macular hole development.

### 2.2. Role of Müller Cells in the Pathogenesis of IMH

#### 2.2.1. Unique Function of Müller Cells

Müller cells are also called radial glial cells. The cell bodies of Müller cells are located in the inner nuclear layer (INL), and many cellular processes in Müller cells span the whole thickness of the neurosensory retina. Müller cells form the inner retinal surfaces at the ILM level, and all types of cell bodies and the retinal neuronal processes are ensheathed in these cells. In contrast, Müller cells proliferate and form the external limiting membrane (ELM) at the PR layer level. The ELM is connected to the ILM at the fovea center due to thickening of the Müller cell layer, creating a cone-shaped appearance. This forms the Müller cap, which is a reservoir of xanthophylls, and it enables Müller cells to protect to the retina [18, 19]. Moreover, multiple functional interactions exist between neurons and Müller cells, including interactions involving glutamate recycling, substance exchange in ganglion cells, and extracellular ion homeostasis, etc. Müller cells possess an abundance of neurotransmitter receptors and voltage-gated channels and are involved in mediate neuronal cell protection [15, 16, 20–22].

#### 2.2.2. Spatial Distribution of Müller Cells in the Fovea of the Macula

*(1) Spatial Distribution of Müller Cells in the Macular Fovea*. Fovea formation involves two major events, i. e., the centrifugal migration of inner retinal cells and the centripetal movement of peripheral PRs. As a result, the fovea lacks the inner retinal layer, and the cones exist in the fovea alone. Gass proposed that Müller cell cones (MCCs), which are structures formed by Müller cells that take the shape of inverted cones, occupy almost of the one-third space of the internal foveola, and the MCCs act as glue in the foveola that bind all cone cells together. Retinal PR cells might perform centrifugal contraction without such a “plug”. The retinal structure of the central foveola, which is considered the weakest part of the fovea, might be more easily affected by anterior vitreofoveal traction [18, 23, 24].

Müller cells in the fovea can be divided into the following 3 types: MCC cells [18], atypical Müller cells (ATMs) [25], and Z-shaped Müller cells [26]. MCC cells are located at the center of the foveola, while ATMs are found in the remaining parts of the foveola, a considerable distance away from the MCCs and approximately 400 mm away from the center of the foveola. The Z-shaped Müller cells are located in the parafoveolar area [18, 24–27].

#### 2.2.3. Involvement of Müller Cells in MH Formation

When considering the special arrangement of Müller cells in the macular fovea and the degree and extent of A-P vitreomacular traction, there are two different types of full-thickness defects of the fovea: the dehiscent type and the tearing type.

The dehiscent type is characterized by cleavage towards the internal margin of the Henle fiber layer and the formation of foveolar pseudocysts, which was accompanied by the centrifugal tangent displacement of the photoreceptor cell layer, and vitreous traction is exerted on the foveolar floor. The smallest loss of the foveolar tissue would be realized if the ATMs and MCCs are pulled, which is especially true in the external layer that includes the PRs. If the intensity or extent of vitreofoveal traction and adhesion is great, then the Z-shaped Müller cells with eccentricity to the central area of the foveolar floor (the clivus) would be affected by this traction force. In the tearing type, the clivus witnesses the separation if the Z-shaped Müller cells is involved, being accompanied with extensive loss of the outer retinal tissue during full-thickness tearing [13, 14, 27–31]. In 1995, Gass modified the previous classification and proposed that the dehiscent-type defect, instead of the tearing type defects, was the origin of most of the MH cases [32].

## 3. Metamorphopsia

### 3.1. Definition and Significance of Metamorphopsia

Metamorphopsia in patients is the perception deviation of the horizontal or vertical line, and it sometimes may appear before the clinical manifestation of macular disease [33–38]. Patients may sometimes consider metamorphopsia as an unstable condition of vision instead of precise deformations of objects, and they focus better with slightly unconscious eye movements. Specific types of metamorphopsia include macropsia and micropsia. Micropsia, in which the human eye perceives an object to be smaller than it actually is, is more common. In macropsia, an object is perceived to be larger than it actually is. Recent studies have reported the nonlinear interactions between the shape and size of visual distortion and recognition of letters. In addition, Wittich et al. confirmed that there is no correlation between metamorphopsia and preoperative visual acuity [39–41]. This finding indicates that it may be difficult to distinguish the change in distortion from the change in acuity. Amsler emphasized that it was important to detect a visual disorder early, although it could not be found in the fundus examination or the standard quantitative test (visual acuity examination, for example) of visual functions.

Visual distortion is the result of the structure change in the retina that causes the retinal layer to suffer displacement, which in turn causes distortion in space. As a common clinical manifestation of different macular diseases, visual distortion can serve as a sensitive biomarker to detect the occurrence, development, and alleviation of macular diseases. Some of the most common clinical manifestations of macular degeneration involving metamorphopsia include retinal detachment, macular serous detachment, vitreoretinal interface disease, CSC (central serous chorioretinopathy), diabetic macular edema and nondiabetic macular edema, and AMD (age-related macular degeneration).

Normal vision and a normal fundus may be present in functional disorders, but macular degeneration may occur. Therefore, in clinical practice, symptoms regarding visual function in patients are often underestimated. Visual distortion also significantly reduces the ability of patients to read and discriminate, and metamorphopsia negatively affects the quality of life of patients [42–45].

### 3.2. Pathogenesis of Metamorphopsia

The development of metamorphopsia involves both the cortical mechanism and the retinal mechanism, despite the previous perception that metamorphopsia was just caused by structural changes to the retina, with outer retinal displacement causing inappropriate optical signal transmission. With the advent of spectral OCT, the detailed re-evaluation of the retina could be performed. As a result, detailed reports of the relationship between metamorphopsia and OCT parameters have emerged, and these reports partially confirmed the previous hypothesis that viewed the change in the PR layers as the major reason for metamorphopsia. Nevertheless, some recent theories emphasize a correlation between the inner retina and cortical processing and metamorphopsia. The cerebral cortex is mainly affected by visual crowding effects and perceptual “filling-in” [24, 30, 33, 46–56].

### 3.3. Method of Measuring Metamorphopsia

To evaluate metamorphopsia, many functional examinations can be conducted, such as the Amsler grid and the mobile handheld home surveillance devices [57]. The Amsler grid [34, 37, 38, 58] was the first functional test to be evaluated for visual distortion. This test enables simple and fast qualitative assessment of changes in the visual function in the field of view of central 10°, which might appear prior to certain macular diseases or together with the development of these diseases. However, the standard Amsler grid, as a suprathreshold trial stimulus, cannot be used to detect early metamorphopsia because of its poor sensitivity; thus various trials have been inspired by the Amsler grid for the application of the quantification and early detection of metamorphopsia. Most of these tests, however, still need full clinical validation. For example, M-CHARTS, differential perimetry, SDH (shape discrimination hyperacuity), and PHP (preferential hyperacuity perimeter) tests have been applied to metamorphopsia assessment [59–62].

#### 3.3.1. Amsler Grid

The Amsler grid is a black piece of cardboard of 10 square centimeters that is divided into small squares of 5 mm with white lines. A white point in the center is used as the fixed point. Black lines on a white background are also useful. When the grid is placed 28–30 cm in front of the patient's eyes, the whole grid is equivalent to 20°, and each small square is equivalent to 1°. This square was therefore designed by Amsler to test for diseases in the range of 10°. The blind spot is located at the nasal boundary approximately 5° outside the grid. Although the Amsler grid has been extensively applied to visual distortion, quantifying the severity of metamorphopsia is still difficult [34, 37, 38, 58].

#### 3.3.2. M-CHARTS

In 1999, Matsumoto et al. [63] proposed quantifying the seriousness of visual distortion using M-CHARTS (Inami Co., Tokyo, Japan). The basic principle of the method is that when the dotted line substitutes for the straight line and the coarse intervals substitute the dot intervals, line distortion is reduced. Furthermore, when the dot intervals are enhanced, line distortion continues to be reduced until the line becomes nearly straight. M-CHARTS includes nineteen dotted lines, and the visual angle of dot intervals ranges from 0.2 degree to 2.0 degrees. Moreover, M-CHARTS is divided into two types: the type 1 single-line M-CHARTS (for general use) and the type 2 double-line M-CHARTS; the type 2 is composed of 2 lines that have an intervening fixation point for the purpose of MH patients. In this test, the patient is first asked to gaze at the target at the center and then vertical straight lines (0°) are shown. If the straight line is perceived as straight by the patient, then the metamorphopsia score of the patient is 0, and the test is complete. In contrast, if the patient sees the straight line as a curve or other irregular shapes, then he/she will be shown many interrupted lines composed of different dots, from fine dots to coarse dots. If the dotted line is recognized as straight by the patient, then the metamorphopsia score of the patient will be determined to be the visual angle used to separate the dots. Next, the same test is conducted using the horizontal lines after rotating the M-CHARTS at 90°. These tests are performed three times, with the mean value applied to data analysis. The advantages of M-CHARTS are its low cost, its portability, and its ease of use. However, it also has disadvantages of not being applicable to patients with serious central dark spots or low vision due to its design purpose of detecting and quantifying subtle deformations and is thus very demanding of visual quality. Nowomiejska et al. made a comparative analysis of the Amsler grid and M-CHARTS in evaluating bevacizumab for the treatment of neovascular AMD in metamorphopsia patients using OCT, and they concluded that the sensitivity of M-CHARTS might be better than that of the Amsler grid [50].

### 3.4. Different Manifestations of Visual Distortion

The Amsler grid can be used to detect a large number of metamorphopsia in different forms. A straight line can break into a small/large wavy line or an angular deformity. In micropsia, the horizontal lines and the vertical lines appear to bend towards the center, while in macropsia, the lines bend from the center. In addition, researchers found correlations between macular diseases and metamorphopsia of varied types. For example, there are associations between small angular defects and myopic maculopathy, between AMD and wavy lines, between retinal detachment and diffuse visual distortions, and between postoperative retinal reattachment and diffuse irregularity of the grid (e.g., depicting by a tremulous hand) [34, 46, 59, 64–66].

## 4. MH and Metamorphopsia

### 4.1. Classification of Metamorphopsia in IMH Patients

In a retrospective study by Saito et al. [67] that included 54 eyes from 51 MH patients, two types of visual distortion were found using the Amsler grid test: the first type was pincushion distortion, with lines curved towards the center, and the second type was unpatterned metamorphopsia without a specific form. A total of 33 cases (61%) of pincushion distortion and 21 cases (39%) of unpatterned metamorphopsia were identified, and an association was found between the stage and duration of MH and the type of metamorphopsia. Approximately 70% of patients with early MH (duration ≤6 months) have pincushion distortion. In a comprehensive study of MH by Gass, it was found that vitreous tangential traction on the retina could enlarge the MH, and the enlargement of the MH is not accompanied by tissue loss [32]. In early MH, displacement of the PR layer caused by retinal shrinkage leads the vertical line to stimulate the displaced PR cells, thus forming the perception of a line curved towards the center, and this is pincushion distortion [68]. The retinal PR layer is most displaced in the central part of the fovea, and the peripheral part is gradually reduced. Patients with a short duration of MH maintain good PR function and have less perceptual filling-in in the brain. The perceptual filling-in effect occurs when a patient observes the Amsler grid, and an area that originally corresponded to the retinal defect is seen as distorted but continuous. One of the general examples of the perceptual filling-in effect is the filling-in of a nonphotosensitive area (physiological blind spot) of the retina which has a partial image in the surrounding area.

Compared with structural changes in the retina, the subjective visual perception of the patient is much more complicated. Patients with long-term MH exhibit unpatterned metamorphopsia. Because the MH is usually surrounded by a fluid cuff that leads to functional retinal damage, patients with long disease courses are more likely than those with short disease courses to lose retinal function around the hole. In addition, patients with long-term MH are more likely than those with short-term MH to lose retinal function surrounding the macular region. If the foveal cells become nonfunctioning during a long-standing case, the patient will have developed a true foveal scotoma. Schuchard noted that the scotoma is usually perceptual filling-in, and such filling-in is often reported by patients as distortion resembling unpatterned metamorphopsia depicted in this paper. Distortion in this case is considered to be the result of the mechanism of the cerebral cortex rather than simple retinal projection [67].

### 4.2. Study on the Mechanism between Metamorphopsia and IMH

Krøyer et al. [69, 70] claimed that the reasons for metamorphopsia were the eversion of PRs (caused by eversion of internal edges of holes) and tangential displacement of the PRs. They speculated that two types of PR layer displacements play a role in the metamorphopsia of MH: tangential displacement and eversion of the outer retina. In pincushion distortion, tangential displacement of PRs and eversion of PRs may explain why the patients perceive the flatter curve farther away from the fovea and the steepest curve nearest to the fovea center, respectively. Eversion of the outer retina is a critical factor for induced metamorphopsia and displacement of the PR layer. According to this theory, surgery may be sufficient to close the hole by eliminating retinal eversion, but metamorphopsia can persist, even after the inner layer of the hole is closed. However, the PR layer retains residual tangential displacement even after the inner layer of the MH has been closed. After successful repair of the MH, residual metamorphopsia unrelated to eccentricity indicates that PR cells are not completely repositioned. As Michalewska et al. described, even after the MH is closed, subretinal cysts still occur in many patients before the PR layer is completely repositioned [71], which explains why metamorphopsia persists after the MH is successfully repaired surgically (in which eversion of the MH edge has been eliminated and closure of the MH has been achieved; however, there is still tangential displacement). Burke concluded that both the cortical visual filling-in effect and PR layer displacement are important factors that can explain the degree of metamorphopsia in MH [72, 73].

### 4.3. Clinical Studies on Metamorphopsia in IMH

Arimura et al. [62] performed a linear regression analysis of the metamorphopsia score (M-score) measured using M-CHARTS and the MH shape (hole size and fluid cuff length). The finding is that there was an important correlation between the M-score and the fluid cuff (superficial retinal detachment), but there was no relationship between the M-score and MH diameter. To be specific, the significant correlation between the fluid cuff and M-score exists when the MH was smaller than 0.5 mm, but if the MH was bigger than 0.5 mm, such a significant correlation would not be present. No significant correlation was found between the M-score and the visual acuity [39]. Sugiura et al. [74] used type I M-CHARTS in a prospective continuous interventional study of 51 eyes and found that the preoperative and postoperative vertical M-scores of the MH patients were significantly higher than the horizontal M-scores. They also found a correlation between the average postoperative M-scores and the length of the ELM defect and the preoperative basal diameter of the MH and the preoperative area of intraretinal cysts in the fluid cuff. The largest influence factor of severity of metamorphopsia is attributed to the preoperative area of intraretinal cysts [39, 41, 57, 75, 76].

In addition, the degree of metamorphopsia obtained using M-CHARTS is correlated with the foveal shape after surgical MH closure, particularly asymmetric extension of the foveal tissue. The reason for this might be the induction of asymmetric tangential traction by asymmetric extension of the foveal tissue to prevent displacement of the PRs during recovery. It is true that asymmetric elongation generally appears in the INL and other nuclear layers instead of the PR layer, but PR displacement can be incurred by the traction of a certain retinal layer, which causes further visual distortion [77]. Another possible explanation is that the correct signal transduction is blocked by asymmetric extension of the retinal layer. In MH, many patients have intraretinal cysts, which may distort retinal microstructures such as Müller cells, amacrine cells, bipolar cells, and horizontal cells. Such morphological changes may cause abnormal synaptic function and reduce the sensitivity of PR cells, causing metamorphopsia. Associations exist between the horizontal M-score and vertical retinal changes, and between the vertical M-score and horizontal retinal changes [65, 72, 78].

Kim et al. [79] reported that tissues stretched longer towards the thicker regions of the retina. It has been speculated that the power of traction in the nasal direction is stronger when MH occurs, causing the horizontal retinal changes to be greater than the vertical retinal changes, which might explain why preoperative horizontal M-scores are smaller than preoperative vertical M-scores. After MH surgery, the fovea asymmetrically extends towards the nasal retina; thus, we speculate that the greater nasal extension provides a higher postoperative vertical metamorphopsia score.

Krasnicki et al. [80] reported improvements in metamorphopsia and BCVA (best-corrected visual acuity) after MH surgery. Considering the EZ (ellipsoid zone) defect and recovery of the ELM, a relatively long period of time was needed for improvement in BCVA to be observed. In contrast, metamorphopsia improved more quickly because postoperative morphology improved after the surgery and then entered a stable stage [71, 75].

Kinoshita et al. [72] reported improvement in horizontal metamorphopsia at 12 months after MH surgery, but vertical metamorphopsia gained stable improvement at 6 months. Okamoto et al. [65] revealed a significant poorer performance of horizontal metamorphopsia at 3–6 months after surgery compared with that at baseline. However, vertical metamorphopsia showed a significant improvement from baseline at 3 months after surgery but did not show a significant improvement at 6 months. Therefore, improvement in postoperative vertical metamorphopsia may not be as notable as improvement in postoperative horizontal metamorphopsia. It is possible that the directionality of retinal plasticity is limited by the existence of the optic disc and the direction of retinal nerve fibers.

## 5. Conclusion

Visual function affects a patient's visual quality of life and is not limited to postoperative visual acuity; postoperative improvement in metamorphopsia is also important. Through an in-depth study of the pathogenesis of MH and metamorphopsia, we conclude that metamorphopsia in MH is the result of tangential centrifugal displacement of the PR cell layer. As the disease progresses, the cerebral cortex participates in the development of metamorphopsia through the perceptual filling-in effect, which is in contrast to the simple abnormal retinal projection hypothesis. Quantitative statistical analysis of metamorphopsia can help determine the prognosis for postoperative visual function in patients, which is of great significance for guiding clinical treatment.
